# Evaluation of fireground exposures using urinary PAH metabolites

**DOI:** 10.1038/s41370-021-00311-x

**Published:** 2021-03-02

**Authors:** Christiane Hoppe-Jones, Stephanie C. Griffin, John J. Gulotta, Darin D. Wallentine, Paul K. Moore, Shawn C. Beitel, Leanne M. Flahr, Jing Zhai, Jin J. Zhou, Sally R. Littau, Devi Dearmon-Moore, Alesia M. Jung, Fernanda Garavito, Shane A. Snyder, Jefferey L. Burgess

**Affiliations:** 1 Department of Chemical and Environmental Engineering, College of Engineering, University of Arizona, Tucson, AZ, USA; 2 Department of Community, Environment and Policy, Mel and Enid Zuckerman College of Public Health, University of Arizona, Tucson, AZ, USA; 3 Tucson Fire Department, Tucson, AZ, USA; 4 Department of Epidemiology and Biostatistics, Mel and Enid Zuckerman College of Public Health, University of Arizona, Tucson, AZ, USA

**Keywords:** Workplace exposures, Polycyclic aromatic hydrocarbons, Cancer, Dermal exposure, Inhalation exposure, Vulnerable occupations

## Abstract

**Background:**

Firefighters have increased cancer incidence and mortality rates compared to the general population, and are exposed to multiple products of combustion including known and suspected carcinogens.

**Objective:**

The study objective was to quantify fire response exposures by role and self-reported exposure risks.

**Methods:**

Urinary hydroxylated metabolites of polycyclic aromatic hydrocarbons (PAH-OHs) were measured at baseline and 2–4 h after structural fires and post-fire surveys were collected.

**Results:**

Baseline urine samples were collected from 242 firefighters. Of these, 141 responded to at least one of 15 structural fires and provided a post-fire urine. Compared with baseline measurements, the mean fold change of post-fire urinary PAH-OHs increased similarly across roles, including captains (2.05 (95% CI 1.59–2.65)), engineers (2.10 (95% CI 1.47–3.05)), firefighters (2.83 (95% CI 2.14–3.71)), and paramedics (1.84 (95% CI 1.33–2.60)). Interior responses, smoke odor on skin, and lack of recent laundering or changing of hoods were significantly associated with increased post-fire urinary PAH-OHs.

**Significance:**

Ambient smoke from the fire represents an exposure hazard for all individuals on the fireground; engineers and paramedics in particular may not be aware of the extent of their exposure. Post-fire surveys identified specific risks associated with increased exposure.

## Introduction

Firefighters in the United States have been shown to have a higher cancer incidence and mortality rate compared with the general population [1]. During fire suppression, firefighters are exposed to multiple chemicals, including but not limited to known and suspected carcinogens such as benzene, formaldehyde, and certain polycyclic aromatic hydrocarbons (PAHs) [2]. As a result, there is a strong desire in the fire service to better characterize and prevent workplace exposures with the objective of reducing cancer risk.

The largest cohort study of firefighters in the United States to date demonstrated an increased rate in lung cancer (10%), gastrointestinal cancer (30–45%), kidney cancer (29%), and mesothelioma (100%) deaths, with similar increases in cancer incidence, compared to the general population [1]. Further analyses demonstrated a significant association between fire hours and increased lung cancer incidence and mortality, along with fire runs and leukemia mortality [3]. In the Australian Firefighters’ Health Study, career male firefighters had significantly elevated prostate cancer, melanoma, and kidney cancer incidence compared to the general Australian population, and a significant increase in lymphohematopoietic cancers associated with duration of service [4]. In this same study, prostate cancer and melanoma incidence were increased in part-time paid firefighters compared to the general population, and among male volunteer firefighters prostate cancer was increased compared to the general population and increased testicular cancer was associated with certain measures of increased exposure. Among female firefighters, there was an increase in colorectal cancer with increasing number of structural fire responses. A study of cancer among firefighters in five Nordic countries revealed a significant excess risk of prostate cancer and melanoma among those 30–49 years of age, as well as an increase in nonmelanoma skin cancer, multiple myeloma, lung adenocarcinoma, and mesothelioma among older firefighters [5]. A previous meta-analysis of 32 studies of cancer in the fire service identified an elevated risk for non-Hodgkin’s lymphoma, prostate cancer, and testicular cancer [6]. These studies consistently found an association between firefighting and cancer, although the specific cancers with elevated rates varied by study.

Fire department policies have traditionally focused on reducing inhalation exposures. Although use of self-contained breathing apparatus (SCBA) greatly reduces the concentration of contaminants that a firefighter inhales, inhalation exposure to carcinogens continues to occur when firefighters are not wearing a SCBA. We previously demonstrated exposures to products of combustion, including carcinogens, during overhaul when historically SCBA were not worn [7, 8]. Furthermore, adverse respiratory effects during overhaul occurred even when air purifying respirators were used [7, 8]. Although use of a SCBA is recommended during overhaul, compliance is not universal, and exposure to smoke may occur during other phases of firefighting as well. In addition, firefighter gear off-gasses detectable levels of benzene, styrene, toluene, xylenes, and other volatile organic chemicals following fireground use [9, 10], potentially contributing to firefighters’ inhalation exposure.

Dermal exposures have been thoroughly documented during firefighting. Wipe samples of skin surfaces collected before and after training fires when firefighters wore their SCBA for all phases including overhaul showed that the neck (protected primarily by Nomex hoods) was the most exposed part of the body [11], and later studies demonstrated high PAH concentrations on the hands of firefighters as well [12]. There is also concern that fireground contaminants can remain in unwashed gear, posing a continuing exposure hazard when reworn [13].

Measurement of urinary hydroxylated metabolites of polycyclic aromatic hydrocarbons (PAH-OHs) concentrations has been used for the assessment of exposure to combustion products in previous studies of firefighters [14, 15]. PAH exposures have been linked to a number of cancers, including skin, lung, bladder, and gastrointestinal cancers [16–18]. Many PAH-related cancers have also been reported at excess rates in firefighters. Among PAHs, known, probable, and possible carcinogens include benzo [*a*]pyrene, dibenz[*a,h*]anthracene, chrysene, benzo[*a*] anthracene, and naphthalene [2, 19, 20]. The current study measured metabolites of naphthalene, fluorene, phenanthrene, and pyrene, the latter three of which are not classifiable as to their carcinogenicity in humans. However, they are measurable in urine after fire exposures and can serve as proxies for the larger mix of PAHs to which firefighters are exposed, including the known carcinogenic PAHs, as well as other products of combustion in smoke and soot. Given their ubiquitous presence in products of combustion, evaluation of PAH metabolites in urine provides a measure of combined inhalation and dermal exposure. As part of a cancer prevention study partnership between the University of Arizona and the Tucson Fire Department (TFD), we set out to evaluate exposure to combustion products through measurement of urinary PAH metabolites in firefighters following structural fires based on their roles in the fire and self-reported activities and exposures.

## Materials and methods

### Study setting

The study was approved by the University of Arizona Institutional Review Board, Protocol #1509137073, and all subjects provided informed consent. The study included collection of blood, buccal cells, and urine during annual medical surveillance examinations or during new recruit training for both incumbent and new recruit firefighters, and collection of urine after a structural fire. For subjects not able to provide a baseline urine during enrollment, it was collected after the post-fire urine sample. Inclusion criteria included being TFD uniformed personnel and responding to fires as part of their current duties. A survey evaluating firefighter demographics, medical and occupational history, and recent exposures, was collected at baseline. A survey evaluating actions at the fireground and recent exposures was collected at the fire scene during firefighter rehabilitation, and a second post-fire survey focusing on additional activities after the fire was completed after return to the station at the time of urine collection.

### Urine collection and analysis

Baseline urine samples were collected throughout the day and transported on ice to University of Arizona laboratory the day of collection. Based on an unpublished pilot study by University of Arizona and TFD evaluating exposure to a training fire with firefighters wearing SCBA at all times within the structure, urinary naphthol metabolite concentrations were found to peak 2–4 h following cessation of exposure. For the current study, post-exposure urine samples were therefore collected 2–4 h post-fire by TFD personnel and transported on ice to University of Arizona laboratory within 24 h. Urine was collected in a 120 mL polypropylene collection cup after providing instructions to the firefighter to wash their hands first, void into the container, and return the resealed collection cup to a research team member for refrigeration until processing. A water control was collected and processed in the same manner as the urine collection for each day of baseline and post-fire collections.

Upon arrival in the laboratory, specific gravity was recorded for each sample using the Atago Refractometer (Model PAL-10S, Cat# 4410, Fisher Scientific). Urine samples were centrifuged at 1900 rpm for 10 min, then 10 mL aliquots of the supernatant were frozen at −20 °C until PAH-OH analysis as previously described [21]. This method was in turn based on a prior publication [22], with slight modifications including the use of urine centrifugation instead of filtration prior to deconjugation. In short, urine was digested with β-Glucuronidase from *Helix pomatia*, and extracted using solid phase extraction. Prior to analysis on the gas chromatography tandem mass spectrometry, samples were derivatized. A surrogate standard mix of the deuterated PAH-OHs containing 1-Hydroxynaphthalene-*d*7, 2-Hydroxyphenanthrene-*d*9, 2-Hydroxyfluorene-*d*9, 1-Hydroxypyrene-*d*9, was added to each sample prior to the extraction.

Detection limits were determined to be 175 ng/L, 100 ng/L, 150 ng/L, and 200 ng/L for each of the naphthols, fluorenols, phenanthrols, and 1-hydroxypyrene, respectively. PAH-OH values were multiplied by a specific gravity factor calculated for each urine sample to correct for renal function and individual hydration levels $\left(\text{SGF}=\frac{1.02-1}{SG-1}\right)$. [23]

### Statistical analysis

Non-detectable PAH-OHs were replaced by half the value of their respective detection limit. The PAH-OH concentrations were natural log-transformed to better fit the normal distribution. Univariate and multivariable analyses were performed using a linear mixed-effects model with random intercept to assess mean differences of log-transformed PAH-OHs between baseline and post-fire stratified by job types. The primary outcome was the sum of all PAH-OHs (naphthols, phenanthrols, fluorenols, and 1-hydroxypyrene), and secondary outcomes included the sum of naphthols (1-naphthol and 2-naphthol), sum of phenanthrols (2-phenanthrol, 4-phenanthrol, and 1-phenanthrol + 3-phenanthrol), sum of fluorenols (2-fluorenol, 3-fluorenol, and 9-fluorenol), along with the individual PAH-OHs. Assessment of model fit was performed by the analysis of residuals. All statistical analyses were performed using R version 3.5.3 (https://www.r-project.org) and Stata MP 14.1 (https://www.stata.com/stata14/). Longitudinal analyses were conducted by the R package lme4 [24] and the multilevel mixed-effects linear regression (xtmixed) function in Stata. A two-sided *p* < 0.05 was considered statistically significant.

Firefighters with baseline and post-fire exposure urine samples who took at least one post-fire survey were included in the survey analysis. Univariate regressions were performed to analyze the association between changes in PAH-OH concentrations from baseline to post-fire and each post-exposure survey question. Specifically, the random intercept model was used to control for the serial correlation of repeated intrasubject observations (e.g., multiple measurements of the same subject). The outcomes were the differences of log-transformed PAH-OHs comparing baseline and post-fire urine samples.

## Results

Subject consenting and baseline urine collection started on 10/6/2015 and continued through 11/21/2017. Post-fire urine collection started on 2/9/2016 and continued through 12/19/2016. During this interval, 242 firefighters provided a baseline urine and 141 of these firefighters provided at least one post-fire urine. Some firefighters provided post-fire samples from more than one fire event (range 2–6 fires). Of the 141 subjects in the study that provided both a baseline and post-fire urine, 83 provided the baseline urine after the post-fire urine. With the exception of one subject for whom the baseline urine was collected 48 h after the post-fire urine, all other subjects had at least a 14-day interval between baseline and post-fire urines. The absolute value of the time span between the baseline and post-fire urine samples averaged 135 days, with a maximum of 543 days.

Characteristics of the participating firefighters are listed in Table 1. Most firefighters were male non-Hispanic whites and over half were less than 40 years of age. In both baseline and post-fire subject groups, 28–31% had a body mass index (BMI) in the obese range and 5–6% were either occasional or regular smokers. Because the firefighters measured at post-fire (*n* = 141) are a subsample of those recruited at baseline, a bootstrap method was used to evaluate whether there were significant differences between the two groups. Out of 100 bootstrap sample replicates with 141 individuals, more than 95% showed that there were no significant differences of gender, race/ethnicity, age, BMI, smoker, and rank distributions. When comparing smokers (*n* = 15, including nine occasional and six regular smokers) to nonsmokers (*n* = 226) at baseline, none of the quantified PAH-OHs were significantly different between the two groups (data not shown).

During the study period, 15 fires were studied (Supplemental Table 1). These fires were predominantly residential, including eleven house fires and one apartment fire. Additionally, three commercial fires were studied, including one church, one business, and one school fire. The duration for each fire, measured as the total time that firefighters were on the scene, ranged between 13 and 120 min. In this study, we used the terms offensive and defensive to refer to the overall fire attack strategy, whereas interior and exterior related to the location of individuals during a fire. Twelve of the fires were fought offensively (i.e. fire attack from inside the structure), two of them started as an offensive response and then switched to defensive (i.e. fire attack from the outside of the structure) and one was purely a defensive response. In the offensive fire attacks, firefighters as well as captains operated inside or outside of the burning structure, or both.

**Fig. 1:**
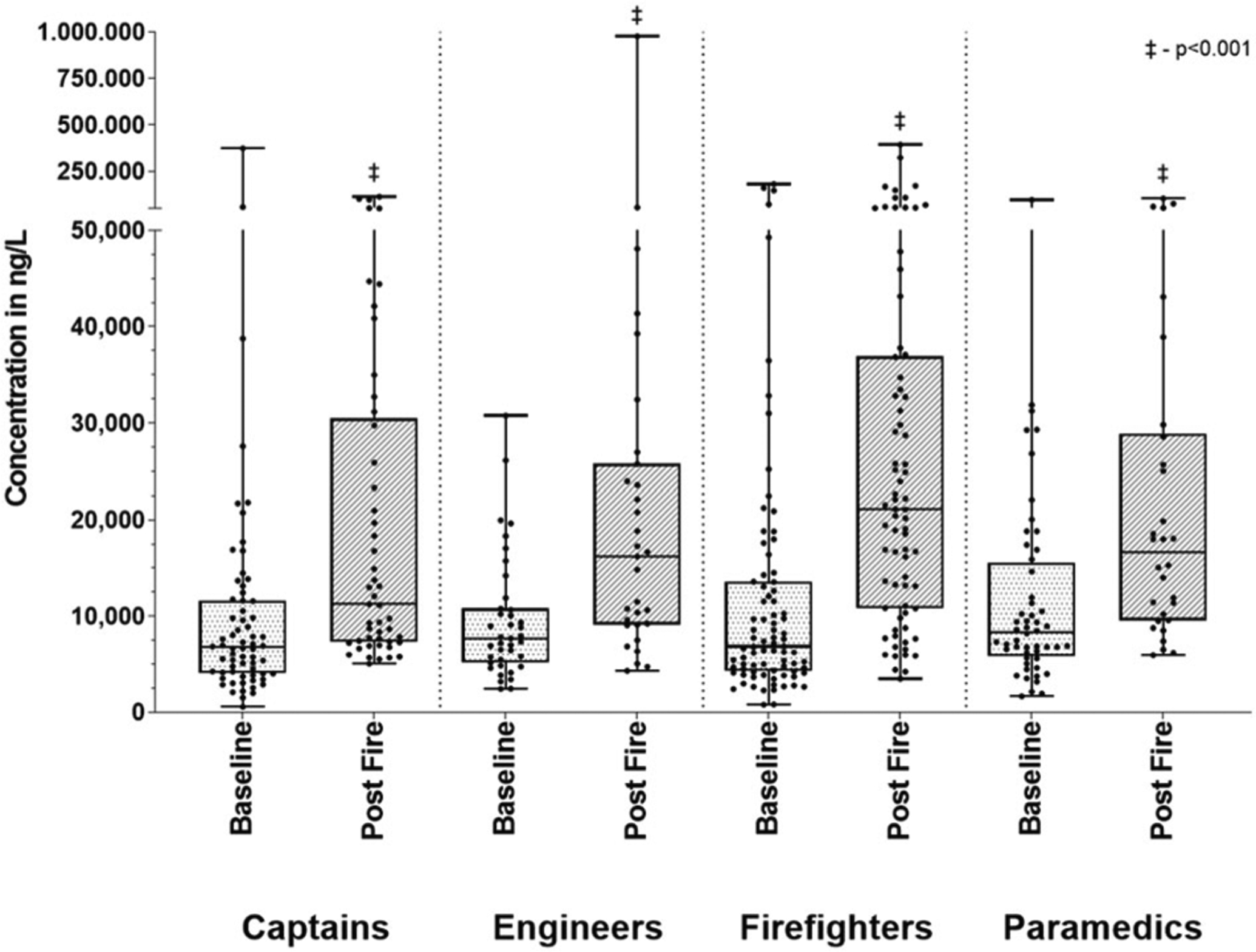
Baseline and post-fire sum of PAH-OHs (including 1-OH-Pyrene) by role in the fire. ‡ - *p* < 0.001.

The concentrations of the sum of urinary PAH-OHs at baseline and post-fire are presented in Table 2 and Fig. 1, categorized by role at the fire. For each PAH-OH, the percent of urine samples with concentrations below the LOD for baseline and post-fire, respectively, varied as follows: 1-naphthol (29%, 3%), 2-naphthol (2%, 0%), 2-fluorenol (83%, 44%), 3-fluorenol (82%, 48%), 9-fluorenol (83%, 44%), 2-phenanthrol (81%, 38%), 4-phenanthrol (97%, 72%), 1-phenanthrol and 3-phenanthrol (52%, 14%) and 1-hydroxypyrene (84%, 51%). Urine specific gravity increased significantly from baseline to post-fire, with means (and 95% confidence intervals) of 1.016 (1.015–1.017) and 1.021 (1.020–1.022), respectively, indicating the firefighters were more dehydrated post-fire. While the statistical analysis was conducted on log-transformed data, the plots were created using the raw data for easier interpretation. All groups (firefighter, captain, engineer, paramedic), with the exception of fire investigators, had a significantly greater (*p* < 0.05) concentration of the sum of urinary PAH-OHs post-fire compared to baseline. The results of multivariable models adjusting for baseline age, BMI, and smoking yielded similar results (data not shown). All groups except the investigators also had significant increases in the sum of naphthols, sum of fluorenols, and sum of phenanthrols comparing baseline and post-fire. Results for individual PAH-OHs are included in the supplementary material (Supplemental Table 2). The sum of urinary PAH-OHs post-fire for each role at individual fires is presented in the supplementary material (Supplemental Fig. 1). Mean post-fire PAH-OH concentrations varied significantly for each fire ranging between 13.196 and 52.422 ng/L.

Evaluation of post-fire survey responses in relation to urinary PAH-OH concentrations is listed in Table 3 for the sum of all PAH-OHs, sum of naphthols, sum of fluorenols, sum of phenanthrols, and 1-hydroxypyrene. Results for individual PAH-OHs are provided in the supplementary material (Supplemental Table 3). Survey response variables associated with the sum of all PAH-OHs in post-fire urines included fire type (commercial vs. residential), interior vs. exterior fire response, duration of interior exposure, smoke odor on the skin, and not having laundered or changed one’s hood within the last month. These same variables were also significantly associated with one or more other urinary PAH-OH markers (sum of naphthols, sum of fluorenols, sum of phenanthrols, and individual PAH-OHs). A number of other survey response variables were not significantly associated with sum of all PAH-OHs but were associated with one or more other PAH-OH markers. These included fire attack minutes, overhaul or salvage minutes, wearing one’s SCBA for over 60% of the time during fire attack or ventilation, and having a dirty hood before the response. Finally, neither total duration of the fire response nor having dirty turnout gear before the response were significantly associated with any of the PAH-OH markers.

## Discussion

Our study demonstrated significant increases in the sum of all urinary PAH-OHs following fireground operations in four main groups: firefighters, captains, engineers, and paramedics. These results also highlight the understudied exposure of engineers and paramedics to combustion emissions while providing nonentry support at fire incidents. Though fire investigators were not found to have a significant increase, only two post-fire urine samples were available for analysis.

The baseline urinary PAH-OH concentrations found in our study are comparable to those of the general population whereas the post-exposure values are less than those seen in the most highly exposed workers. Compared to urinary PAH-OH levels of participants 18–65 years of age in the 2015–2016 National Health and Nutrition Examination Survey (NHANES) multiplied by 1.48 to transform their creatinine corrected values to our specific gravity corrected values [25], the median values for the sum of naphthols in our study (Table 2) were slightly lower than in NHANES (median 12,530 ng/L, IQR 6451–23,918 ng/L). While the sum of phenanthrols were very similar in both studies (NHANES median 357 ng/L, IQR 233–572 ng/L), the median concentrations for the sum of fluorenols and 1-hydroxypyrene were below the detection limit in our study and 357 and 193 ng/L, respectively in the NHANES study. For the sum of all PAH-OHs, the median values and IQR in our study were slightly lower than NHANES (median 13,535 ng/L, IQR 7438–26,106 ng/L), but the analytical method used in our study differed from NHANES and contained two compounds, 9-Fluorenol and 4-Phenanthrol, that were not included in NHANES. The mean post-fire urinary PAH-OHs in our study were at lower concentrations than those reported for coke over workers (2-naphthol 100,000–150,000 ng/L after transformation of the data using a factor of 1.48 to convert creatinine normalized result to specific gravity normalized results as described above) [26]. It should also be noted that coke oven workers are exposed to PAHs for longer durations than firefighters.

Our study results are consistent with other recent studies of firefighter entry teams [14, 15]. Ottawa firefighters responding to fires in the community showed urinary PAH-OH increases from baseline to post-fire of 2.9–5.3 fold depending on the PAH-OH group [14]. This was of similar magnitude to the firefighters in our study, increasing between 3.1 and 5.1 fold for the sum of naphthols, sum of fluorenols and sum of phenanthrols. Although the Ottawa study did not break out the study results by fire response roles, our study found lesser fold increases in the other fire response roles (captains, engineers, paramedics, and investigators) than in firefighters. Differences in study methods included collection of urine over an 18-h period post-fire in the Ottawa study, as compared with our 2–4-h post-fire sampling period. A study of controlled residential fires measured urinary PAH metabolites at 3-h post-fire, similar to our study, although they used a standardized house fire model which was much more consistent both in materials burned and size of the structure across fires than the community responses measured in our study [15]. Nevertheless, their fold increases of 2.4–6.6 based on PAH metabolite group were similar to the 3.1–5.1 fold increased seen in ours. Interestingly their smallest fold increase was for fluorenols, while this group showed the largest fold increase in our study, which could potentially be due to differences in the relative amounts of materials being burned and/or fire and smoke conditions. The controlled residential fire study also found increased urinary PAH-OHs in interior as compared to transitional fire attack (although statistically significant only for the fluorenols), generally consistent with our findings based on self-reported interior v. exterior fire response activities with statistically significant increases for all PAH-OH groups except 1-hydroxypyrene.

The marked variability in post-fire urinary PAH-OH concentrations observed in our study is likely due to differences in exposures based on distinct job tasks within roles at a fire, the complex and evolving nature of each individual fire and differences in use of respiratory protection. Entry/fire attack or ventilation teams are made up of two firefighters and a captain serving as team lead. At times the captain sets up the fireground/tactical operations as the firefighters make the initial entry before the captain joins the firefighters inside the burning structure or on the roof. In addition, we have identified instances where captains removed their respirators to facilitate radio communication while outside of the burning structure but still in a smoky area [27]. This increased exposure may explain why captains had the greatest exposure in 6 of the 15 fires evaluated, as shown in Fig. 1. Other captains may also have roles that require them to stay exterior to the fire. The engineers (also known as driver-operators) work the vehicle pump panel and carry out other outside support activities. The paramedics do not engage directly in firefighting activities but work outside the immediate vicinity of the fire to set up a rehabilitation station for the other fireground personnel. In fires where an engineer or a paramedic had the highest exposure, it is likely that the smoke plume moved over their location after their initial set-up. Although the number of fire cause investigators in the study was limited, they were the group most likely to have measureable 1-hydroxypyrene in their baseline urine samples, and we were not able to exclude the possibility that these levels were from prior fire responses, given the relatively longer elimination half-live of pyrene metabolites compared to the other PAH-OH measured. From an inhalation perspective, the use of respiratory protection while outside of a burning structure varied greatly among study participants. While positive-pressure SCBAs should provide adequate protection against inhalation exposures [28, 29], reduced use of SCBA is common during nonentry fireground activities, particularly during overhaul and activities where the equipment might interfere with mobility and field visibility [30, 31]. Dermal exposure is known to be an important source of PAH absorption as well, as PAH metabolites have been detected in urine of firefighters using SCBA during fire suppression events [11]. The firefighter’s role during the fire event is linked to PAH dermal concentration, with tasks such as fire attack and search correlating to higher contamination on the skin [12, 14]. Both inhalation and dermal exposures need to be considered when planning exposure reduction interventions.

The survey data revealed information which could be used to inform a firefighter job exposure matrix. Response to residential fires had increased exposures compared to commercial fires, although there was a great deal of variability within each group and the number of commercial fires was limited to three. A study of controlled experimental fires found a significant increase in median concentration of urinary PAH-OH metabolites from pre- to post- exposure on firefighters assigned to attack and search roles [15]. In our study, interior response was associated with a substantial increase in concentration of urinary PAH-OHs. This was also supported by the significant association of the number of minutes of interior response with the increase in concentration of urinary PAH-OHs from baseline to post-fire. Contrary to our expectations, overall duration (minutes) of fire response, and minutes of overhaul/salvage were not associated with increase in urinary PAH-OHs. These combined study findings suggest that more detailed exposure records are needed for epidemiologic studies of firefighters, and that the use of cumulative fire hours or fire runs as proxy measures of exposure may need to be refined to include information on interior and exterior responses.

The use of PPE while conducting certain duties was assessed using the surveys to identify its contribution to decreasing exposure. A significant decrease in urinary 2-naphthol and 1- and 3- phenanthrol were observed in individuals who had their SCBA on greater than 60 percent of the time during fire attack and ventilation, respectively, in comparison to those individuals reporting SCBA use 60 percent or less of the time. These results are consistent with other research findings that increased use of SCBA while at a fire scene can decrease exposure to products of combustion [11, 28]. Our ability to evaluate the effectiveness of SCBA use during incident command, pump operation, rapid intervention crew, or emergency medical services was limited due to the small number of individuals wearing their SCBA during these activities.

The survey data also identified that some self-reported exposures were associated with increased urinary PAH-OHs, including smoke odor on the skin. While these results are not surprising, we are not aware that they have been previously studied. A finding contrary to our expectations was that cleaning of the skin with water or a wipe while still on scene was associated with a significant increase in the sum of PAH-OHs. This finding could potentially be explained by a greater use of wipes when exposures had been higher, such as having visible soot on the skin. Dermal decontamination with wipes has been shown to reduce the amount of skin contamination [12, 21], and we previously found that ‘wash-down’ of turnout gear prior to doffing after a fire response, in combination with other fireground interventions, was associated with a 36% reduction in post-fire urinary PAH-OHs [27]. The current practice among many fire departments of hood exchange after a fire is validated by the finding within our study that a longer time interval without cleaning is associated with increased urinary PAH-OHs. In addition, routinely laundered hoods were previously found to have an 81 percent average lower concentration of PAHs compared to unlaundered hoods [32]. There was no significant increase in urinary PAH-OHs associated with wearing turnout gear that had not been recently laundered, although cleaning of turnout gear after each response is considered a best practice [13].

The results of this study affirm the need for fireground exposure reduction interventions for firefighters. Previous studies have investigated various post-fire interventions conducted at the fire scene to reduce dermal and inhalation exposure. Interventions include decontamination of PPE with soap and water along with bagging of gear to reduce exposure from off gassing contaminants, along with cleaning of skin as soon as possible with wipes to reduce dermal absorption [9, 12, 21, 33]. Resources already available to departments recommend the use of these and additional interventions, such as showering and changing of clothes as soon as possible after a fire, having two sets of turnout gear, and diesel capture and removal systems, along with strategies on how to best communicate these interventions for the greatest chance of implementation [13, 34]. The results of this current fireground exposure study were used by the TFD to plan specific exposure reduction interventions, the results of which were previously reported [27].

Limitations of our study include exposure monitoring limited to PAH metabolites, as many other toxic chemicals are present in fire smoke. While this limited scope of exposure monitoring does not affect the study findings, the differential exposures identified may not be generalizable to chemical exposures beyond PAHs. The timing of urine collection at 2–4 h post-exposure was chosen both to maximize the urinary concentrations of 1- and 2- naphthol, as the combined naphthols had the highest concentration of the measured PAH metabolite groups, and because collection at this time period was acceptable by the fire service. However, measurement at this time period likely underestimates post-fire concentrations of PAH-OHs with a longer elimination half-life. The baseline urine was not collected immediately before the fireground response as this was not possible given the unpredictable timing of the fires and the rapid firefighter response to the fires. The urinary PAH-OH concentrations may have been influenced by exposures outside of the fireground, as we did not have any restriction on diet or smoking, both of which can contribute PAH exposures. Beyond smoke from fires, diesel exhaust is also a source of PAH exposure [35], which is associated with acute inflammatory effects [36] and lung and esophageal cancer [37]. Diesel exhaust continues to be an inhalation hazard for firefighters at incident scenes, and also in fire stations, where emissions from the truck bay may infiltrate the living quarters through open doors, cracks in the building, and due to poor ventilation and differences in air pressure between the bays and the living areas [38–40]. We were not able to differentiate between fire smoke and diesel exhaust exposure at the fire scene. Finally, the study was limited to exposure monitoring and the toxicity of the combined exposures was not evaluated.

In conclusion, our study results showed that all fire service personnel at a fire scene are at risk for exposure to products of combustion. Characteristics of the fire, firefighter activities at the scene and self-reported exposures were all significantly associated with urinary sum of all PAH-OHs measurements. Specifically, residential fires, interior responses including duration of interior response, smoke odor on skin, and lack of recent laundering or changing of hoods were significantly associated with increased post-fire urinary sum of PAH-OHs. Fire departments should continue to implement measures to reduce dermal and respiratory exposures.

## Supplementary Material

Supplementary Information for: Evaluation of Fireground Exposures Using Urinary PAH Metabolites

## Figures and Tables

**Table 1:** Study subjects.

Variable	Baseline *n* (%)	Post-fire *n* (%)
Gender		
Male	234 (96.7)	138 (97.9)
Female	8 (3.3)	3 (2.1)
Race/Ethnicity		
White non-Hispanic	206 (85.1)	115 (81.6)
Hispanic	30 (12.4)	22 (15.6)
African American	4 (1.7)	3 (2.1)
Missing	2 (0.8)	1 (0.7)
Age		
<30	33 (13.6)	26 (18.4)
30–40	89 (36.8)	52 (36.9)
≥40	120 (49.6)	62 (44.0)
BMI		
Normal (18.5–25)	31 (12.8)	17 (12.1)
Overweight (25–30)	143 (59.1)	81 (57.4)
Obese (>30)	68 (28.1)	43 (30.5)
Smoker		
No use	226 (93.4)	134 (95.0)
Occasional	9 (3.7)	5 (3.6)
Regular	6 (2.5)	2 (1.4)
Missing	1 (0.4)	0 (0.0)
Rank		
Captain	66 (26.4)	49 (26.2)
Engineer	42 (16.8)	31 (16.6)
Firefighter	84 (33.6)	74 (39.6)
Paramedic	54 (22.6)	30 (16.0)
Investigator	3 (1.2)	2 (1.1)
Missing		1 (0.5)

**Table 2:** Urinary PAH-OHs (ng/L) at baseline and post-fire by role in fire responses.

	FC (95% CI)	Baseline *n*	Post-fire *n*	Baseline median (IQR)	Post-fire median (IQR)
Sum of PAH-OHs^a^					
Captain	**2.06 (1.61–2.64)** ^‡^	66	49	6855 (4216, 11,701)	12,170 (7640, 29,845)
Engineer	**2.08 (1.46–3.02)** ^‡^	39	31	7858 (5402, 11,245)	16,305 (9330, 25,528)
Firefighter	**2.80 (2.13–3.66)** ^‡^	82	74	7380 (4441, 13,744)	21,343 (11,392, 36,415)
Paramedic	**1.84 (1.33–2.59)** ^‡^	52	30	8434 (6156, 15,039)	16,935 (9832, 29,200)
Investigator	1.64 (1.26–2.13)	3	2	4875, 5128, 6935^b^	9595, 11,360^b^
Sum of naphthols					
Captain	**2.03 (1.53–2.71)** ^‡^	66	49	6254 (3528, 10,332)	10,265 (6020, 24,205)
Engineer	**2.05 (1.43–3.01)** ^‡^	39	31	6200 (4436, 9950)	14,800 (7828, 21,640)
Firefighter	**2.91 (2.14–3.94)** ^‡^	82	74	5949 (3636, 12,588)	18,963 (9581, 34,916)
Paramedic	**2.00 (1.32–3.10)** ^†^	52	30	7959 (4945, 11,223)	13,795 (8435, 25,325)
Investigator	1.70 (1.19–4.08)	3	2	2505, 4088, 5945^b^	8530, 10,720^b^
Sum of fluorenols					
Captain	**1.88 (1.44–2.44)** ^‡^	66	49	<LOD (<LOD, <LOD)	315 (<LOD, 895)
Engineer	**2.44 (1.59–3.73)** ^‡^	39	31	<LOD (<LOD, 190)	495 (<LOD, 858)
Firefighter	**3.69 (2.86–4.77)** ^‡^	82	74	<LOD (<LOD, 284)	760 (401, 1510)
Paramedic	**2.49 (1.66–3.76)** ^‡^	52	30	<LOD (<LOD, 419)	798 (346, 1469)
Investigator	1.14 (0.29–3.89)	3	2	<LOD, <LOD, 575^b^	<LOD, 400^b^
Sum of phenanthrols					
Captain	**2.44 (1.83–3.24)** ^‡^	66	49	<LOD (<LOD, 570)	890 (455, 1610)
Engineer	**2.60 (1.71–3.94)** ^‡^	39	31	385 (<LOD, 662)	1055 (548, 1720)
Firefighter	**3.39 (2.62–4.39)** ^‡^	82	73	378 (<LOD, 662)	1180 (805, 2498)
Paramedic	**2.96 (2.13–4.14)** ^‡^	52	30	<LOD (<LOD, 665)	880 (730, 2390)
Investigator	1.93 (0.35–3.54)	3	2	225, 790, 1470^b^	390, 565^b^
1-Hydroxypyrene					
Captain	**1.83 (1.33–2.49)** ^‡^	66	49	<LOD (<LOD, <LOD)	<LOD (<LOD, 685)
Engineer	1.58 (1.00–2.51)	39	31	<LOD (<LOD, <LOD)	<LOD (<LOD, 551)
Firefighter	**2.04 (1.54–2.70)** ^‡^	82	73	<LOD (<LOD, <LOD)	243 (<LOD, 882)
Paramedic	**2.34 (1.57–3.53)** ^‡^	52	30	<LOD (<LOD, <LOD)	<LOD (<LOD, 811)
Investigator	–	3	2	<LOD, 325, 615^b^	<LOD, <LOD

*FC* fold change, *CI* confidence interval.

* *p* < 0.05;

† *p* < 0.01;

‡ *p* < 0.001.

a Includes all naphthols, phenanthrols, fluorenols and 1-hxdroxypyrene.

b Actual values.

Bolded values are statistically significant.

**Table 3:** Multilevel mixed-effects linear regression modeling of urinary PAH-OH fold changes post-fire based on survey responses.

		Sum of PAH-OHs^a^	Sum of naphthols	Sum of phenanthrols	Sum of fluorenols	1-Hydroxypyrene
Variable	*n* (total)	FC (95% CI)	FC (95% CI)	FC (95% CI)	FC (95% CI)	FC (95% CI)
Fire and response type						
Commercial fire (Ref: Residential)	19 (180)	**0.646 (0.421, 0.992)** *	**0.569 (0.356, 0.909)** *	0.836 (0.513, 1.363)	0.949 (0.568, 1.584)	1.142 (0.645, 2.020)
Interior response (Ref: Exterior response)	71 (178)	**1.469 (1.068, 2.021)** *	**1.509 (1.058, 2.153)** *	**1.481 (1.053, 2.083)** *	**1.631 (1.156, 2.302)** ^†^	1.216 (0.820, 1.802)
Duration (minutes)						
Total duration of fire response	180 (180)	1.002 (0.999, 1.006)	1.002 (0.999, 1.006)	1.003 (0.999, 1.006)	0.999 (0.995, 1.003)	1.001 (0.997, 1.006)
Interior response	105 (105)	**1.010 (1.002, 1.019)** *	**1.011 (1.002, 1.020)** *	**1.014 (1.006, 1.023)** ^†^	**1.010 (1.001, 1.019)** *	1.005 (0.994, 1.017)
Fire attack	88 (88)	1.004 (0.995, 1.014)	1.004 (0.994, 1.015)	**1.011 (1.000, 1.022)** *	1.010 (0.999, 1.020)	1.007 (0.995, 1.019)
Overhaul/Salvage	56 (56)	0.999 (0.992, 1.006)	0.999 (0.991, 1.006)	1.000 (0.994, 1.006)	0.998 (0.992, 1.004)	**1.008 (1.001, 1.015)** *
Percent time on air						
Fire attack 61–100% (Ref: 0–60%)	81 (88)	0.527 (0.217, 1.280)	0.393 (0.151, 1.020)	1.122 (0.433, 2.907)	1.030 (0.400, 2.656)	0.851 (0.329, 2.203)
Overhaul/Salvage 61–100% (Ref: 0–60%)	48 (56)	0.949 (0.400, 2.253)	1.004 (0.375, 2.684)	1.001 (0.481, 2.082)	1.094 (0.493, 2.430)	0.821 (0.336, 2.004)
Ventilation 61–100% (Ref: 0–60%)	21 (23)	0.668 (0.254, 1.756)	0.730 (0.266, 2.002)	0.250 (0.052, 1.202)	1.163 (0.352, 3.839)	1.561 (0.193, 12.64)
Rehab 61–100% (Ref: 0–60%)	3 (25)	1.792 (0.695, 4.620)	1.709 (0.572, 5.105)	1.454 (0.361, 5.859)	0.865 (0.269, 2.779)	0.766 (0.141, 4.166)
Odor, soot, and gear status						
Smoke odor on skin (Ref: No)	43 (180)	**1.461 (1.032, 2.066)** *	1.442 (0.980,2.123)	1.299 (0.885, 1.906)	1.060 (0.712, 1.577)	1.026 (0.659, 1.597)
Black mucus in nose, mouth or throat (Ref: No)	20 (46)	1.010 (0.471, 2.168)	0.982 (0.433, 2.228)	1.420 (0.920, 2.192)	1.135 (0.501, 2.568)	0.614 (0.290, 1.298)
Washed with water or wipe on scene (Ref: No)	30 (46)	**2.095 (1.021, 4.299)** *	2.226 (0.998, 4.963)	**2.073 (1.883, 2.283)** ^†^	2.227 (0.965, 5.141)	0.639 (0.288, 1.419)
Turnout gear dirty (soot) before response (Ref: No)	83 (180)	1.013 (0.732, 1.403)	1.073 (0.748, 1.542)	0.781 (0.554, 1.100)	0.948 (0.667, 1.348)	1.025 (0.693, 1.516)
Hood dirty (soot) before response (Ref: No)	27 (171)	1.437 (0.903, 2.285)	1.357 (0.808, 2.279)	1.416 (0.870,2.306)	**1.688 (1.040, 2.738)** *	1.546 (0.892, 2.677)
Laundered turnout gear >1 month (Ref: <1 mo)	117 (180)	1.172 (0.863, 1.592)	1.238 (0.882, 1.738)	0.966 (0.690, 1.352)	0.977 (0.690, 1.384)	0.771 (0.525, 1.131)
Laundered/changed hood >1 month (Ref: <1 mo)	92 (180)	**1.468 (1.108, 1.945)** *	**1.581 (1.160, 2.155)** ^†^	1.234 (0.898, 1.697)	1.067 (0.767, 1.486)	0.774 (0.536, 1.119)

*FC* fold change, *CI* confidence interval.

* *p* < 0.05;

† *p* < 0.01.

a Includes all naphthols, phenanthrols, fluorenols, and 1-hxdroxypyrene.

Bolded values are statistically significant.
