# Supplementary material for: Evaluation of fireground exposures using urinary PAH metabolites
Source: J Expo Sci Environ Epidemiol. Author manuscript; Available in PMC 2021 Sep 18. (PMC8445814; doi:10.1038/s41370-021-00311-x)
Supplement: Supplementary Information for: Evaluation of Fireground Exposures Using Urinary PAH Metabolites [file NIHMS1739748-supplement-Supplementary_Information_for__Evaluation_of_Fireground_Exposures_Using_Urinary_PAH_Metabolites.pdf]

Supplementary Information for: Evaluation of Fireground Exposures Using Urinary PAH Metabolites

Christiane Hoppe-Jones, PhD; Stephanie C. Griffin, PhD; John J. Gulotta; Darin D. Wallentine; Paul K. Moore; Shawn C. Beitel, MS; Leanne M. Flahr, MS; Jing Zhai, PhD; Jin J. Zhou, PhD; Sally R. Littau; Devi Dearmon-Moore; Alesia M. Jung, MS; Fernanda Garavito, MPH; Shane A. Snyder, PhD; Jefferey L. Burgess, MD, MS, MPH

Affiliations: Department of Chemical and Environmental Engineering, College of Engineering, University of Arizona, Tucson, Arizona (Dr. Hoppe-Jones, Mr. Beitel, Ms. Flahr, Dr. Snyder); Department of Community, Environment and Policy, Mel and Enid Zuckerman College of Public Health, University of Arizona, Tucson, Arizona (Dr. Burgess, Dr. Griffin, Ms. Littau, Ms. Dearmon-Moore, Ms. Garavito); Tucson Fire Department, Tucson, Arizona (Mr. Gulotta, Mr. Wallentine, Mr. Moore); Department of Epidemiology and Biostatistics, Mel and Enid Zuckerman College of Public Health, University of Arizona, Tucson, Arizona (Dr. Zhou, Dr. Zhai, Ms. Jung).

Address correspondence to: Jefferey L. Burgess, MD, MS, MPH, Mel and Enid Zuckerman College of Public Health, University of Arizona, 1295 N Martin Ave, Tucson, AZ 85724; Fax number (520) 626-6093; Telephone number (520) 626-4918; Email address jburgess@email.arizona.edu

Included in this file:

Supplementary Figure 1

Supplementary Tables 1-3

Table of Contents

Supplemental Figure 1. Post-fire sum of urinary PAH-OHs by fire and role in the fire……. 3

Supplemental Table 1. Description of fires………………………………………………… 4

Supplemental Table 2. Individual PAH-OH measurements at baseline and post-fire by job classification………………………………………………………………………………… 5

Supplemental Table 3. Post-fire survey responses relative to urinary naphthol and phenanthrol metabolites (ng/L)…………………………………………………………………………… 7

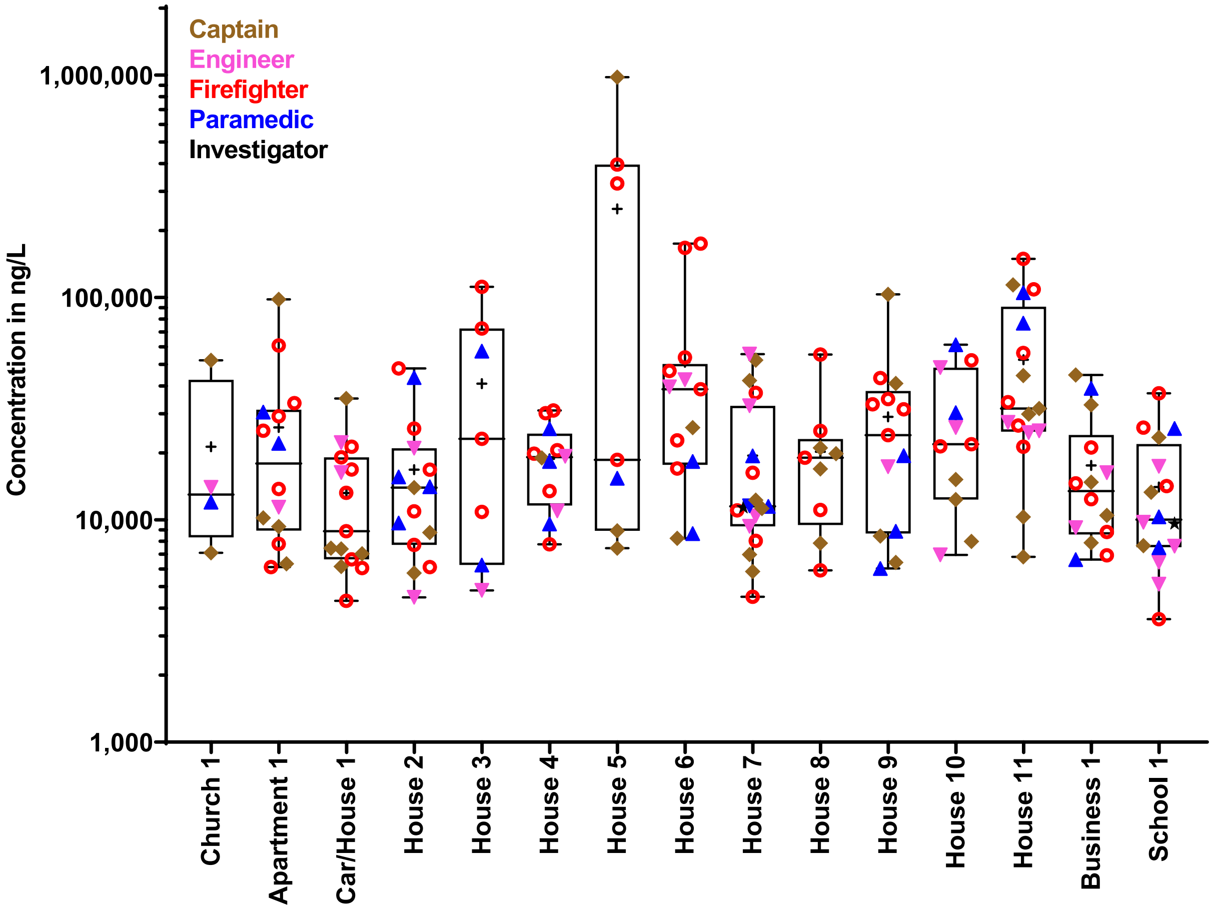

Supplemental Figure 1. Post-fire sum of urinary PAH-OHs by fire and role in the fire.

(+ signifies the mean concentration for each fire)

Supplementary Table 1. Description of Fires

**Identifier**	**Duration**	**Offensive/Defensive***	**Number of post-fire urines collected†**	**Time into fire (military time)‡**
Church 1	33 min	Offensive	4	02:59
Apartment 1	19 min	Offensive	14	15:05
Car/House 1	21 min	Offensive	15	07:00
House 2	40 min	Offensive	16	18:51
House 3	40 min	Offensive	9	16:06
House 4	18 min	Offensive	13	20:55
House 5	34 min	Offensive; went defensive halfway	7	11:14
House 6	43 min	Offensive	13	10:35
House 7	21 min	Offensive; went defensive halfway	19	08:46
House 8	13 min	Offensive	9	14:56
House 9	18 min	Offensive	13	10:13
House 10	41 min	Defensive	11	13:06
House 11	120 min	Offensive	17	12:38
Business 1	43 min	Offensive	14	18:58
School 1	20 min	Offensive	16	04:42

* An offensive fire attack is from the interior of the structure and a defensive fire attack is from the outside of the structure; † For each fire one post-fire urine sample was collected from each firefighter study participant, but additional firefighters not in the study also responded to the fire; ‡ Time into fire refers to the time of day that firefighters arrived on the fire scene.

Supplemental Table 2. Individual PAH-OH measurements at baseline and post-fire by job classification.

	FC (95% CI)	Baseline n	Post-Fire n	Baseline mean (SD)	Frequency of detection (Baseline)	Post-fire mean (SD)	Frequency of detection (Post-fire)
**1-Naphthol**							
Captain	**4.05 (2.59-6.34)‡**	66	49	1,197 (2,695)	46/66	3,193 (4,211)	48/49
Engineer	**4.49 (2.44-8.24)‡**	39	31	1,086 (1,577)	31/39	22,233 (104,618)	31/31
Firefighter	**6.81 (4.44-10.40)‡**	82	74	1,196 (2,578)	58/82	9,677 (25,302)	73/74
Paramedic	**3.75 (1.97-7.12)‡**	52	30	1,147 (1,825)	37/52	4,849 (8,617)	27/30
Investigator	1.68 (0.99-3.73)	3	2	568 (467)	2/3	1,693	2/2
**2-Naphthol**							
Captain	**2.05(1.46-2.86)‡**	66	49	12,867 (43,600)	64/66	17,266 (21,592)	49/49
Engineer	**1.79 (1.31-2.49)‡**	39	31	7,656 (5,877)	39/39	23,159 (57,415)	31/31
Firefighter	**2.70 (1.97-3.68)‡**	82	74	13,961 (29,696)	80/82	25,891 (35,981)	74/74
Paramedic	**1.87 (1.21-2.94)†**	52	30	10,112 (13,137)	50/52	16,112 (13,584)	30/30
Investigator	1.74 (1.03-5.92)	3	2	3612 (1545)	3/3	7933	2/2
**2-Fluorenol**							
Captain	**1.99 (1.53-2.57)‡**	66	49	75 (76)	8/66	179 (169)	23/49
Engineer	**2.83 (1.82-4.40)‡**	39	31	87 (107)	6/39	444 (969)	19/31
Firefighter	**3.57 (2.63-4.84)‡**	82	74	95 (96)	20/82	614 (1,174)	51/74
Paramedic	**3.38 (2.30-5.04)‡**	52	30	92 (99)	10/52	459 (544)	22/30
Investigator	1.14 (0.22-5.96)	3	2	120 (121)	1/3	123	1/2
**3-Fluorenol**							
Captain	**1.49 (1.17-1.91)***	66	49	90 (161)	10/66	133 (121)	20/49
Engineer	**1.67 (1.14-2.44)***	39	31	74 (63)	6/39	231 (479)	12/31
Firefighter	**2.48 (1.90-3.23)‡**	82	74	86 (107)	16/82	314 (434)	43/74
Paramedic	**2.29 (1.59-3.31)‡**	52	30	143 (429)	10/52	247 (206)	20/30
Investigator	1.24 (0.18-5.57)	3	2	138 (153)	1/3	103	1/2
**9-Fluorenol**							
Captain	**1.80 (1.31-2.47)‡**	66	49	76 (86)	7/66	256 (465)	17/49
Engineer	**2.61 (1.57-4.31)‡**	39	30	85 (125)	3/39	929 (3,599)	15/30
Firefighter	**4.29 (3.20-5.75)‡**	79	73	72 (85)	10/79	584 (1,214)	52/73
Paramedic	**2.19 (1.31-3.65)†**	51	30	243 (525)	15/51	419 (522)	19/30
Investigator	--	2	2	--	0/2	--	0/2

**1-and 3-Phenanthrol**							
Captain	**2.27 (1.56-3.29)‡**	66	49	221 (224)	26/66	603 (684)	35/49
Engineer	**2.65 (1.58-4.19)‡**	39	31	281 (345)	22/39	947 (1,735)	27/31
Firefighter	**4.03 (3.05-5.31)‡**	82	73	214 (199)	44/82	1,108 (1,738)	66/73
Paramedic	**3.23 (2.19-4.80)‡**	52	30	293 (400)	22/52	962 (1,188)	29/30
Investigator	--	3	2	--	2/3	--	2/2
**2-Phenanthrol**							
Captain	**3.11 (2.19-4.40)‡**	66	49	167 (203)	17/66	640 (759)	36/49
Engineer	**3.60 (2.18-5.95)‡**	39	30	190 (260)	13/39	1,314 (3,621)	24/30
Firefighter	**4.23 (3.06-5.85)‡**	82	73	227 (287)	34/82	1,280 (2,479)	64/73
Paramedic	**4.27 (2.80-6.64)‡**	52	30	311 (1,135)	15/52	1,024 (1,296)	27/30
Investigator	1.28 (0.15-6.53)	3	2	243 (292)	1/3	163	1/2
**4-Phenanthrol**							
Captain	**1.22 (1.08-1.39)†**	66	49	75 (0)	0/66	113 (119)	7/49
Engineer	1.49 (1.10-2.04)	39	31	82 (32)	2/39	298 (876)	9/31
Firefighter	**1.61 (1.29-1.99)‡**	82	73	96 (120)	5/82	251 (529)	28/73
Paramedic	**1.44 (1.18-1.77)‡**	52	30	75 (0)	0/52	169 (220)	7/30
Investigator	--	3	2	--	1/3	--	0/2
**1-Hydroxypyrene**							
Captain	**1.83 (1.33-2.49)‡**	66	49	195 (339)	8/66	403 (475)	23/49
Engineer	1.58 (1.00-2.51)	39	30	263 (391)	9/39	486 (664)	14/30
Firefighter	**2.04 (1.54-2.70)‡**	82	74	212 (294)	17/82	625 (841)	39/74
Paramedic	**2.34 (1.57-3.53)‡**	52	30	257 (1077)	3/52	636 (938)	14/30
Investigator	--	3	2	--	2/3	--	0/2

* $p<0.05$; † $p<0.01$; ‡ $p<0.001$; -- no results available due to lack of statistical model convergence

Supplemental Table 3. Post-fire survey responses relative to urinary naphthol and phenanthrol metabolites (ng/L).

	n (total N)	1- Naphthol	2-Naphthol	n	1- and 3-Phenanthrol	4- Phenanthrol	2-Phenanthrol
Variable		Coefficient (95% CI)	Coefficient (95% CI)		Coefficient (95% CI)	Coefficient (95% CI)	Coefficient (95% CI)
Fire and response type							
Commercial (Ref: Residential)	19 (180)	0.698 (0.331, 1.472)	**0.550 (0.347, 0.871)***	19	0.697 (0.405, 1.199)	0.846 (0.577, 1.240)	1.030 (0.553, 1.922)
Interior response (Ref: No)	71 (180)	**2.965 (1.750, 5.023)†**	1.322 (0.925, 1.891)	71	**1.615 (1.101, 2.369)***	**1.408 (1.108, 1.789)†**	1.374 (0.894, 2.112)
Duration (minutes)							
Total duration of fire response	180 (180)	1.000 (0.994, 1.006)	1.003 (0.999, 1.006)	180	1.004 (0.999, 1.008)	1.000 (0.997, 1.003)	1.003 (0.998, 1.008)
Interior response	105 (105)	**1.019 (1.006, 1.033)†**	**1.009 (1.001, 1.017)***	105	**1.013 (1.004, 1.023)†**	**1.014 (1.005, 1.022)†**	**1.018 (1.007, 1.030)†**
Fire attack	88 (88)	1.011 (0.993, 1.029)	1.003 (0.994, 1.013)	88	1.011 (0.999, 1.023)	1.005 (0.995, 1.015)	**1.015 (1.001, 1.028)***
Overhaul/Salvage	56 (56)	1.005 (0.994, 1.016)	0.999 (0.990, 1.007)	56	1.003 (0.997, 1.010)	0.999 (0.994, 1.003)	0.996 (0.988, 1.004)
Percent time on air							
Fire attack >60% (Ref: ≤60%)	81 (88)	1.353 (0.311, 5.893)	**0.380 (0.154, 0.936)***	81	0.983 (0.357, 2.707)	0.998 (0.458, 2.174)	1.025 (0.312, 3.371)
Overhaul/salvage >60% (Ref: ≤60%)	48 (56)	0.780 (0.193, 3.151)	0.973 (0.344, 2.750)	48	0.633 (0.271, 1.479)	--	1.979 (0.724, 5.409)
Ventilation >60% (Ref: ≤60%)	21 (23)	1.164 (0.139, 9.778)	0.698 (0.264, 1.841)	21	**0.120 (0.017, 0.846)***	--	0.374 (0.071, 1.963)
Rehab >60% (Ref: ≤60%)	3 (25)	2.669 (0.427, 16.69)	1.590 (0.906, 2.790)	3	0.997 (0.145, 6.844)	2.179 (0.948, 5.011)	1.622 (0.415, 6.333)
Odor, soot and gear status							
Smoke Odor on Skin (Ref: No)	43 (180)	1.627 (0.892, 2.966)	1.330 (0.903, 1.958)	43	**1.641 (1.072, 2.513)***	1.223 (0.925, 1.618)	0.948 (0.582, 1.543)
Black mucus in nose, mouth or throat (Ref: No)	20 (46)	2.028 (0.652, 6.310)	0.911 (0.411, 2.020)	20	1.823 (0.895, 3.715)	1.190 (0.724, 1.956)	1.268 (0.627, 2.562)
Washed with water or wipe on scene (Ref: No)	30 (46)	**3.408 (1.121, 10.36)***	1.807 (0.802, 4.070)	30	**2.637 (1.435, 4.845)†**	**1.740 (1.111, 2.724)***	1.456 (0.670, 3.162)
Turnout gear dirty (soot) before response (Ref: No)	83 (180)	0.977 (0.561, 1.701)	1.046 (0.728, 1.505)	83	0.837 (0.568, 1.233)	0.852 (0.669, 1.084)	0.696 (0.453, 1.068)
Hood dirty (soot) before response (Ref: No)	27 (171)	1.743 (0.787, 3.858)	1.295 (0.770, 2.178)	27	1.477 (0.855, 2.552)	1.272 (0.905, 1.788)	1.319 (0.717, 2.426)
Laundered turnout gear >1 month (Ref: <1 month)	117 (180)	1.103 (0.650, 1.871)	1.296 (0.927, 1.810)	117	0.852 (0.584, 1.242)	1.081 (0.842, 1.387)	0.908 (0.594, 1.389)
Laundered/changed hood >1 month (Ref: <1 mo)	92 (180)	1.553 (0.945, 2.550)	**1.604 (1.182, 2.176)†**	92	1.192 (0.834, 1.704)	1.230 (0.971, 1.558)	1.154 (0.768, 1.734)

* p<0.05; † p<0.01; ‡ Most recent laundering; -- no results available due to lack of statistical model convergence
