# Supplementary material for: Evaluation of fireground exposures using urinary PAH metabolites
Source: J Expo Sci Environ Epidemiol. 2021 Mar 2;31(5):913–22. doi: 10.1038/s41370-021-00311-x (PMC8445814; doi:10.1038/s41370-021-00311-x)
Supplement: Supplementary file 1 [file 41370_2021_311_MOESM1_ESM.docx]

**Supplementary Information for: Evaluation of Fireground Exposures Using Urinary PAH Metabolites**

Christiane Hoppe-Jones, PhD; Stephanie C. Griffin, PhD; John J. Gulotta; Darin D. Wallentine; Paul K. Moore; Shawn C. Beitel, MS; Leanne M. Flahr, MS; Jing Zhai, PhD; Jin J. Zhou, PhD; Sally R. Littau; Devi Dearmon-Moore; Alesia M. Jung, MS; Fernanda Garavito, MPH; Shane A. Snyder, PhD; Jefferey L. Burgess, MD, MS, MPH

**Affiliations**: Department of Chemical and Environmental Engineering, College of Engineering, University of Arizona, Tucson, Arizona (Dr. Hoppe-Jones, Mr. Beitel, Ms. Flahr, Dr. Snyder); Department of Community, Environment and Policy, Mel and Enid Zuckerman College of Public Health, University of Arizona, Tucson, Arizona (Dr. Burgess, Dr. Griffin, Ms. Littau, Ms. Dearmon-Moore, Ms. Garavito); Tucson Fire Department, Tucson, Arizona (Mr. Gulotta, Mr. Wallentine, Mr. Moore); Department of Epidemiology and Biostatistics, Mel and Enid Zuckerman College of Public Health, University of Arizona, Tucson, Arizona (Dr. Zhou, Dr. Zhai, Ms. Jung).

**Address correspondence to**: Jefferey L. Burgess, MD, MS, MPH, Mel and Enid Zuckerman College of Public Health, University of Arizona, 1295 N Martin Ave, Tucson, AZ 85724; Fax number (520) 626-6093; Telephone number (520) 626-4918; Email address [jburgess@email.arizona.edu](mailto:jburgess@email.arizona.edu)

**Included in this file:**

**Supplementary Figure 1**

**Supplementary Tables 1-3**

**Table of Contents**

**Supplemental Figure 1.** Post-fire sum of urinary PAH-OHs by fire and role in the fire…….	3

**Supplemental Table 1.** Description of fires……………………………………………………	4

**Supplemental Table 2.** Individual PAH-OH measurements at baseline and post-fire by job classification……………………………………………………………………………………	5

**Supplemental Table 3.** Post-fire survey responses relative to urinary naphthol and phenanthrol metabolites (ng/L)………………………………………………………………………………	7

Supplemental Figure 1. Post-fire sum of urinary PAH-OHs by fire and role in the fire.

(+ signifies the mean concentration for each fire)

Supplementary Table 1. Description of Fires

| **Identifier** | **Duration** | **Offensive/Defensive*** | **Number of post-fire urines collected**^**†**^ | **Time into fire (military time)**^**‡**^ |
| --- | --- | --- | --- | --- |
| Church 1 | 33 min | Offensive | 4 | 02:59 |
| Apartment 1 | 19 min | Offensive | 14 | 15:05 |
| Car/House 1 | 21 min | Offensive | 15 | 07:00 |
| House 2 | 40 min | Offensive | 16 | 18:51 |
| House 3 | 40 min | Offensive | 9 | 16:06 |
| House 4 | 18 min | Offensive | 13 | 20:55 |
| House 5 | 34 min | Offensive; went defensive halfway | 7 | 11:14 |
| House 6 | 43 min | Offensive | 13 | 10:35 |
| House 7 | 21 min | Offensive; went defensive halfway | 19 | 08:46 |
| House 8 | 13 min | Offensive | 9 | 14:56 |
| House 9 | 18 min | Offensive | 13 | 10:13 |
| House 10 | 41 min | Defensive | 11 | 13:06 |
| House 11 | 120 min | Offensive | 17 | 12:38 |
| Business 1 | 43 min | Offensive | 14 | 18:58 |
| School 1 | 20 min | Offensive | 16 | 04:42 |

* An offensive fire attack is from the interior of the structure and a defensive fire attack is from the outside of the structure; ^**†**^ For each fire one post-fire urine sample was collected from each firefighter study participant, but additional firefighters not in the study also responded to the fire; ^**‡**^ Time into fire refers to the time of day that firefighters arrived on the fire scene. 

Supplemental Table 2. Individual PAH-OH measurements at baseline and post-fire by job classification.

|  | **FC (95% CI)** | **Baseline n** | **Post-Fire n** | **Baseline mean (SD)** | **Frequency of** **detection (Baseline)** | **Post-fire mean (SD)** | **Frequency of** **detection (Post-fire)** |
| --- | --- | --- | --- | --- | --- | --- | --- |
| **1-Naphthol** |  |  |  |  |  |  |  |
| Captain | **4.05 (2.59-6.34)‡** | 66 | 49 | 1,197 (2,695) | 46/66 | 3,193 (4,211) | 48/49 |
| Engineer | **4.49 (2.44-8.24)‡** | 39 | 31 | 1,086 (1,577) | 31/39 | 22,233 (104,618) | 31/31 |
| Firefighter | **6.81 (4.44-10.40)‡** | 82 | 74 | 1,196 (2,578) | 58/82 | 9,677 (25,302) | 73/74 |
| Paramedic | **3.75 (1.97-7.12)‡** | 52 | 30 | 1,147 (1,825) | 37/52 | 4,849 (8,617) | 27/30 |
| Investigator | 1.68 (0.99-3.73) | 3 | 2 | 568 (467) | 2/3 | 1,693 | 2/2 |
|  |  |  |  |  |  |  |  |
| **2-Naphthol** |  |  |  |  |  |  |  |
| Captain | **2.05(1.46-2.86)‡** | 66 | 49 | 12,867 (43,600) | 64/66 | 17,266 (21,592) | 49/49 |
| Engineer | **1.79 (1.31-2.49)‡** | 39 | 31 | 7,656 (5,877) | 39/39 | 23,159 (57,415) | 31/31 |
| Firefighter | **2.70 (1.97-3.68)‡** | 82 | 74 | 13,961 (29,696) | 80/82 | 25,891 (35,981) | 74/74 |
| Paramedic | **1.87 (1.21-2.94)†** | 52 | 30 | 10,112 (13,137) | 50/52 | 16,112 (13,584) | 30/30 |
| Investigator | 1.74 (1.03-5.92) | 3 | 2 | 3612 (1545) | 3/3 | 7933 | 2/2 |
|  |  |  |  |  |  |  |  |
| **2-Fluorenol** |  |  |  |  |  |  |  |
| Captain | **1.99 (1.53-2.57)‡** | 66 | 49 | 75 (76) | 8/66 | 179 (169) | 23/49 |
| Engineer | **2.83 (1.82-4.40)‡** | 39 | 31 | 87 (107) | 6/39 | 444 (969) | 19/31 |
| Firefighter | **3.57 (2.63-4.84)‡** | 82 | 74 | 95 (96) | 20/82 | 614 (1,174) | 51/74 |
| Paramedic | **3.38 (2.30-5.04)‡** | 52 | 30 | 92 (99) | 10/52 | 459 (544) | 22/30 |
| Investigator | 1.14 (0.22-5.96) | 3 | 2 | 120 (121) | 1/3 | 123 | 1/2 |
|  |  |  |  |  |  |  |  |
| **3-Fluorenol** |  |  |  |  |  |  |  |
| Captain | **1.49 (1.17-1.91)*** | 66 | 49 | 90 (161) | 10/66 | 133 (121) | 20/49 |
| Engineer | **1.67 (1.14-2.44)*** | 39 | 31 | 74 (63) | 6/39 | 231 (479) | 12/31 |
| Firefighter | **2.48 (1.90-3.23)‡** | 82 | 74 | 86 (107) | 16/82 | 314 (434) | 43/74 |
| Paramedic | **2.29 (1.59-3.31)‡** | 52 | 30 | 143 (429) | 10/52 | 247 (206) | 20/30 |
| Investigator | 1.24 (0.18-5.57) | 3 | 2 | 138 (153) | 1/3 | 103 | 1/2 |
|  |  |  |  |  |  |  |  |
| **9-Fluorenol** |  |  |  |  |  |  |  |
| Captain | **1.80 (1.31-2.47)‡** | 66 | 49 | 76 (86) | 7/66 | 256 (465) | 17/49 |
| Engineer | **2.61 (1.57-4.31)‡** | 39 | 30 | 85 (125) | 3/39 | 929 (3,599) | 15/30 |
| Firefighter | **4.29 (3.20-5.75)‡** | 79 | 73 | 72 (85) | 10/79 | 584 (1,214) | 52/73 |
| Paramedic | **2.19 (1.31-3.65)†** | 51 | 30 | 243 (525) | 15/51 | 419 (522) | 19/30 |
| Investigator | -- | 2 | 2 | -- | 0/2 | -- | 0/2 |
|  |  |  |  |  |  |  |  |
| **1-and 3-Phenanthrol** |  |  |  |  |  |  |  |
| Captain | **2.27 (1.56-3.29)‡** | 66 | 49 | 221 (224) | 26/66 | 603 (684) | 35/49 |
| Engineer | **2.65 (1.58-4.19)‡** | 39 | 31 | 281 (345) | 22/39 | 947 (1,735) | 27/31 |
| Firefighter | **4.03 (3.05-5.31)‡** | 82 | 73 | 214 (199) | 44/82 | 1,108 (1,738) | 66/73 |
| Paramedic | **3.23 (2.19-4.80)‡** | 52 | 30 | 293 (400) | 22/52 | 962 (1,188) | 29/30 |
| Investigator | -- | 3 | 2 | -- | 2/3 | -- | 2/2 |
|  |  |  |  |  |  |  |  |
| **2-Phenanthrol** |  |  |  |  |  |  |  |
| Captain | **3.11 (2.19-4.40)‡** | 66 | 49 | 167 (203) | 17/66 | 640 (759) | 36/49 |
| Engineer | **3.60 (2.18-5.95)‡** | 39 | 30 | 190 (260) | 13/39 | 1,314 (3,621) | 24/30 |
| Firefighter | **4.23 (3.06-5.85)‡** | 82 | 73 | 227 (287) | 34/82 | 1,280 (2,479) | 64/73 |
| Paramedic | **4.27 (2.80-6.64)‡** | 52 | 30 | 311 (1,135) | 15/52 | 1,024 (1,296) | 27/30 |
| Investigator | 1.28 (0.15-6.53) | 3 | 2 | 243 (292) | 1/3 | 163 | 1/2 |
|  |  |  |  |  |  |  |  |
| **4-Phenanthrol** |  |  |  |  |  |  |  |
| Captain | **1.22 (1.08-1.39)†** | 66 | 49 | 75 (0) | 0/66 | 113 (119) | 7/49 |
| Engineer | 1.49 (1.10-2.04) | 39 | 31 | 82 (32) | 2/39 | 298 (876) | 9/31 |
| Firefighter | **1.61 (1.29-1.99)‡** | 82 | 73 | 96 (120) | 5/82 | 251 (529) | 28/73 |
| Paramedic | **1.44 (1.18-1.77)‡** | 52 | 30 | 75 (0) | 0/52 | 169 (220) | 7/30 |
| Investigator | -- | 3 | 2 | -- | 1/3 | -- | 0/2 |
|  |  |  |  |  |  |  |  |
| **1-Hydroxypyrene** |  |  |  |  |  |  |  |
| Captain | **1.83 (1.33-2.49)‡** | 66 | 49 | 195 (339) | 8/66 | 403 (475) | 23/49 |
| Engineer | 1.58 (1.00-2.51) | 39 | 30 | 263 (391) | 9/39 | 486 (664) | 14/30 |
| Firefighter | **2.04 (1.54-2.70)‡** | 82 | 74 | 212 (294) | 17/82 | 625 (841) | 39/74 |
| Paramedic | **2.34 (1.57-3.53)‡** | 52 | 30 | 257 (1077) | 3/52 | 636 (938) | 14/30 |
| Investigator | -- | 3 | 2 | -- | 2/3 | -- | 0/2 |

* p<0.05; † p<0.01; ‡ p<0.001; -- no results available due to lack of statistical model convergence

Supplemental Table 3. Post-fire survey responses relative to urinary naphthol and phenanthrol metabolites (ng/L).

|  | n (total N) | 1- Naphthol | 2-Naphthol | n | 1- and 3-Phenanthrol | 4- Phenanthrol | 2-Phenanthrol |
| --- | --- | --- | --- | --- | --- | --- | --- |
| Variable |  | Coefficient (95% CI) | Coefficient (95% CI) |  | Coefficient (95% CI) | Coefficient (95% CI) | Coefficient (95% CI) |
| Fire and response type |  |  |  |  |  |  |  |
| Commercial (Ref: Residential) | 19 (180) | 0.698 (0.331, 1.472) | **0.550 (0.347, 0.871)*** | 19 | 0.697 (0.405, 1.199) | 0.846 (0.577, 1.240) | 1.030 (0.553, 1.922) |
| Interior response (Ref: No) | 71 (180) | **2.965 (1.750, 5.023)**† | 1.322 (0.925, 1.891) | 71 | **1.615 (1.101, 2.369)*** | **1.408 (1.108, 1.789)**† | 1.374 (0.894, 2.112) |
| Duration (minutes) |  |  |  |  |  |  |  |
| Total duration of fire response | 180 (180) | 1.000 (0.994, 1.006) | 1.003 (0.999, 1.006) | 180 | 1.004 (0.999, 1.008) | 1.000 (0.997, 1.003) | 1.003 (0.998, 1.008) |
| Interior response | 105 (105) | **1.019 (1.006, 1.033)**† | **1.009 (1.001, 1.017)*** | 105 | **1.013 (1.004, 1.023)**† | **1.014 (1.005, 1.022)**† | **1.018 (1.007, 1.030)†** |
| Fire attack | 88 (88) | 1.011 (0.993, 1.029) | 1.003 (0.994, 1.013) | 88 | 1.011 (0.999, 1.023) | 1.005 (0.995, 1.015) | **1.015 (1.001, 1.028)*** |
| Overhaul/Salvage | 56 (56) | 1.005 (0.994, 1.016) | 0.999 (0.990, 1.007) | 56 | 1.003 (0.997, 1.010) | 0.999 (0.994, 1.003) | 0.996 (0.988, 1.004) |
| Percent time on air |  |  |  |  |  |  |  |
| Fire attack >60% (Ref: ≤60%) | 81 (88) | 1.353 (0.311, 5.893) | **0.380 (0.154, 0.936)*** | 81 | 0.983 (0.357, 2.707) | 0.998 (0.458, 2.174) | 1.025 (0.312, 3.371) |
| Overhaul/salvage >60% (Ref: ≤60%) | 48 (56) | 0.780 (0.193, 3.151) | 0.973 (0.344, 2.750) | 48 | 0.633 (0.271, 1.479) | -- | 1.979 (0.724, 5.409) |
| Ventilation >60% (Ref: ≤60%) | 21 (23) | 1.164 (0.139, 9.778) | 0.698 (0.264, 1.841) | 21 | **0.120 (0.017, 0.846)*** | -- | 0.374 (0.071, 1.963) |
| Rehab >60% (Ref: ≤60%) | 3 (25) | 2.669 (0.427, 16.69) | 1.590 (0.906, 2.790) | 3 | 0.997 (0.145, 6.844) | 2.179 (0.948, 5.011) | 1.622 (0.415, 6.333) |
| Odor, soot and gear status |  |  |  |  |  |  |  |
| Smoke Odor on Skin (Ref: No) | 43 (180) | 1.627 (0.892, 2.966) | 1.330 (0.903, 1.958) | 43 | **1.641 (1.072, 2.513)*** | 1.223 (0.925, 1.618) | 0.948 (0.582, 1.543) |
| Black mucus in nose, mouth or throat (Ref: No) | 20 (46) | 2.028 (0.652, 6.310) | 0.911 (0.411, 2.020) | 20 | 1.823 (0.895, 3.715) | 1.190 (0.724, 1.956) | 1.268 (0.627, 2.562) |
| Washed with water or wipe on scene (Ref: No) | 30 (46) | **3.408 (1.121, 10.36)*** | 1.807 (0.802, 4.070) | 30 | **2.637 (1.435, 4.845)**† | **1.740 (1.111, 2.724)*** | 1.456 (0.670, 3.162) |
| Turnout gear dirty (soot) before response (Ref: No) | 83 (180) | 0.977 (0.561, 1.701) | 1.046 (0.728, 1.505) | 83 | 0.837 (0.568, 1.233) | 0.852 (0.669, 1.084) | 0.696 (0.453, 1.068) |
| Hood dirty (soot) before response (Ref: No) | 27 (171) | 1.743 (0.787, 3.858) | 1.295 (0.770, 2.178) | 27 | 1.477 (0.855, 2.552) | 1.272 (0.905, 1.788) | 1.319 (0.717, 2.426) |
| Laundered turnout gear >1 month (Ref: <1 month) | 117 (180) | 1.103 (0.650, 1.871) | 1.296 (0.927, 1.810) | 117 | 0.852 (0.584, 1.242) | 1.081 (0.842, 1.387) | 0.908 (0.594, 1.389) |
| Laundered/changed hood >1 month (Ref: <1 mo) | 92 (180) | 1.553 (0.945, 2.550) | **1.604 (1.182, 2.176)**† | 92 | 1.192 (0.834, 1.704) | 1.230 (0.971, 1.558) | 1.154 (0.768, 1.734) |

* p<0.05; † p<0.01; ‡ Most recent laundering; -- no results available due to lack of statistical model convergence
